# Does the effect of flowering time on biomass allocation across latitudes differ between invasive and native salt marsh grass *Spartina alterniflora*?

**DOI:** 10.1002/ece3.8681

**Published:** 2022-03-07

**Authors:** Wenwen Liu, Xincong Chen, Jiayu Wang, Yihui Zhang

**Affiliations:** ^1^ 12466 Key Laboratory of the Ministry of Education for Coastal and Wetland Ecosystems College of the Environment and Ecology Xiamen University Fujian China

**Keywords:** biomass allocation, evolution, flowering time, latitude, salt marsh

## Abstract

1. Parallel latitudinal clines in flowering time have been documented in both the invasive and native ranges of plants. Furthermore, flowering time has been found to affect biomass at maturity. Therefore, understanding how these flowering times affect biomass accumulation across latitudes is essential to understanding plant adaptations and distributions.
2. We investigated and compared trends in first flowering day (FFD), aboveground biomass (AGB), belowground biomass (BGB), and BGB:AGB ratio of the salt marsh grass *Spartina alterniflora* along latitudinal gradients from the invasive (China, 19–40°N) and native range (United States, 27–43°N) in a greenhouse common garden experiment, and tested whether FFD would drive these divergences between invasive and native ranges.
3. The invasive populations produced more (~20%, ~19%) AGB and BGB than native populations, but there were no significant differences in the FFD and BGB:AGB ratio. We found significant parallel latitudinal clines in FFD in both invasive and native ranges. In addition, the BGB:AGB ratio was negatively correlated with the FFD in both the invasive and native ranges but nonsignificant in invasive populations. In contrast, AGB and BGB increased with latitude in the invasive range, but declined with latitude in the native range. Most interestingly, we found AGB and BGB positively correlated with the FFD in the native range, but no significant relationships in the invasive range.
4. Our results indirectly support the evolution of increased competitive ability hypothesis (EICA) that *S*. *alterniflora* has evolved to produce greater AGB and BGB in China, but the flowering and allocation pattern of native populations is maintained in the invasive range. Our results also suggest that invasive *S*. *alterniflora* in China is not constrained by the trade‐off of earlier flowering with smaller size, and that flowering time has played an important role in biomass allocation across latitudes.

## Graphical abstract

How flowering time affects biomass accumulation across latitudes is essential to understanding plant adaptations and distributions. We found AGB and BGB positively correlated with the FFD in the native range, but no significant relationships in the invasive range, indicating that invasive *Spartina alterniflora* in China is not constrained by the commonly observed life history trade‐off of earlier flowering with smaller size at flowering.

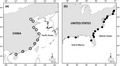

## 1 INTRODUCTION

Biological invasions offer an opportunity to study how the evolution of flowering time affects vegetative growth during colonization across space and time (Helliwell et al., 2018; Hodgins et al., 2018; van Kleunen et al., 2018). Invasive plants might evolve to flower earlier (McGoey et al., 2020), and to grow faster, larger, and with greater biomass compared to natives (Colautti et al., 2009; Hanley, 2012; Hodgins & Rieseberg, 2011; Shang et al., 2015; Sobrinho et al., 2013). Life‐history theory predicts that there is a positively genetic correlation between flowering time with plant size and biomass, selection for earlier flowering will result in maturation of smaller individuals due to physiological trade‐offs (Stearns, 1989). Accordingly, plant size has strongly positive correlation with shoot biomass (Liu & Pennings, 2019), thus flowering time has a positive effect on plant size and biomass at maturity (Colautti & Barrett, 2013; Helsen et al., 2020; Li et al., 2015; Montague et al., 2008). When there is sufficient genetic variation, the genotypes that flower earlier at a larger size would be favored, because the evolution of earlier flowering significantly increases the fitness of invasive plants and facilitated their range expansions (Colautti & Barrett, 2013). Therefore, the phenotypic traits variation in most plants may result primarily from changes in the flowering time (Galloway & Burgess, 2012).

Geographic gradients in environmental conditions can select for clinal adaptation in various traits, a phenomenon that has been demonstrated in many invasive plant species (Donohue, 2017; Etterson et al., 2016; Kollmann & Banuelos, 2004; Ridley & Ellstrand, 2010). Additionally, clines in traits of invasive and native populations offer an opportunity to study parallel evolution, and whether phenotypic variation is due to phenotypic plasticity or local adaptation (Alexander et al., 2012; van Boheemen et al., 2019; Hodgins et al., 2018; Maron et al., 2004; McGoey et al., 2020). However, the interplay of local adaptation and plasticity in driving latitudinal variation in biomass allocation is rare (Castillo et al., 2014, 2018; Zenni et al., 2014).

Reproductive timing is an important adaptive transition from vegetative to reproductive growth across latitudinal gradients (Barrett et al., 2008; Colautti & Barrett, 2010; Griffith & Watson, 2005, 2006), where populations at higher latitudes often evolve to flower earlier to ensure successful reproduction in shorter growing seasons, resulting in a clinal pattern of plant size and flowering time (Griffith & Watson, 2006; Haggerty & Galloway, 2011; Santamaria et al., 2003). Biomass allocations are expected to reflect a functional balance between the distribution of resources that exist above and below the soil surface (Franklin et al., 2012), and shifts in above‐ versus belowground resource availability may alter relative plant investments in root biomass (Ma et al., 2021). Although most studies have compared flowering phenology and biomass allocation between invasive and native ranges of species (Beckmann et al., 2009; Blossey & Nötzold, 1995; Brown & Eckert, 2005; Hanley, 2012; Meyer & Hull‐Sanders, 2008; Sobrinho et al., 2013), there are few empirical studies comparing the effect of flowering time on biomass allocation across latitudes between invasive and native ranges (Liu et al., 2022; McGoey et al., 2020; Sun & Roderick, 2019).

Salt marshes are highly productive ecosystems and one of the most valuable carbon sinks on the planet, providing ecosystem services with provisioning, supporting, regulating, and cultural services and economic benefits (Barbier et al., 2011; McLeod et al., 2011; Townend et al., 2011). The productivity of salt marsh plants plays a key role in these services (Kirwan & Murray, 2007). Moreover, highly aboveground biomass and belowground biomass can accumulate large amounts of sediments against the effects of sea level rise and storms (Kirwan & Megonigal, 2013). Thus, production of plants in marsh ecosystems is important both for carbon sequestration and the persistence of marshes with rising sea level (Kirwan & Mudd, 2012). Above‐ and belowground biomass and biomass allocation provide a foundation for better understanding ecosystem structure and function in salt marshes (Crosby et al., 2017). Salt marsh plants allocated relatively more biomass belowground at higher latitude to withstand freezing and store carbon reserves belowground over the winter, but allocated relatively more biomass aboveground at low latitude because the plants do not experience prolonged freezing (Crosby et al., 2017; Kirwan et al., 2009). Overall, the allocation of biomass is a key adaptive strategy for salt marsh plants that could enhance carbon‐sequestration capacity and stability under global climate change.

*Spartina alterniflora* is native to the United States (27–45°N) (Kirwan et al., 2009; Strong & Ayres, 2013). Since its introduction into China in 1979, this species is now widely distributed in the intertidal marshes with regular tidal flooding from 19°N to 40°N latitude (An et al., 2007; Liu et al., 2016; Xu & Zhuo, 1985). This has been the largest and most recent of many substantial invasions of *S*. *alterniflora* around the world (Strong & Ayres, 2013). Previous studies have reported latitudinal variation in growth, reproduction, or defense of *S*. *alterniflora* in its invasive and native ranges (Liu, Chen, et al., 2020; Liu, Zhang, et al., 2020; Strong & Ayres, 2013). The aboveground biomass of *S*. *alterniflora* decreases with latitude in its native range (Kirwan et al., 2009), which appears to have a genetic basis (Liu, Chen, et al., 2020; Liu, Zhang, et al., 2020). Biomass allocation notably changes between high and low latitudes, which decreased allocation to belowground biomass with increasing latitude (Crosby et al., 2017). However, the aboveground biomass of *S*. *alterniflora* showed a hump‐shaped relationship with latitude in its invasive range in China, which is mainly due to phenotypic plasticity (Liu, Chen, et al., 2020; Liu et al., 2016, 2017; Liu, Zhang, et al., 2020). Furthermore, most research of biomass allocation was conducted at local scales, focused on the response to some abiotic factors (Castillo et al., 2008; Darby & Turner, 2008; Snedden et al., 2015). However, the broader scale pattern of this latitudinal variation in biomass accumulation, and its relationship with flowering time, is still unknown (but see Crosby et al., 2015).

Here, we ask whether biomass allocation variation in *S*. *alterniflora* along latitudes has a genetic basis or is driven by phenotypic plasticity. If so, we ask whether biomass genetic differentiation among populations was dependent on flowering time. Therefore, we compared first flowering day, aboveground biomass, belowground biomass, and belowground biomass:aboveground biomass ratio of *S*. *alterniflora* from the invasive and native ranges in a greenhouse common garden experiment. Through this, we address the following questions: (1) do invasive populations exhibit earlier flowering phenology and greater biomass compared with native populations? (2) do flowering time and biomass allocation vary along latitudes and does the response to latitudes differ between ranges? (3) do the relationships between flowering time and aboveground biomass, belowground biomass, and aboveground versus belowground biomass allocation differ between ranges?

## 2 MATERIALS AND METHODS

### 2.1 Study locations and seed collections

The peak seed production lasted from September to November throughout the coastal area of China in 2014 (Chen et al., 2021). We collected seeds at 10 locations spanning 20° of latitude from 20.9° (Guangdong, province) to 39.0°N (Tianjin, province) in China (Figure 1a) in September‐November, 2014. We also collected seeds at 16 locations spanning 16° of latitude from 27.7° (Florida) to 43°N (Maine) in the United States (Figure 1b) in October and November 2014. At each location, we worked at two sites, 2–3 km apart. At each site, we sampled five 0.5 × 0.5 m quadrats, with at least 30 m spacing between quadrats, each quadrat was treated as a seed family. We randomly collected 10 inflorescences within a meter of each quadrat. We collected the filled seeds in each inflorescence (Daehler & Strong, 1994; Liu et al., 2016). Filled seeds have an embryo, endosperm, and can potentially germinate and grow; unfilled seeds have neither of these tissues and cannot germinate or grow (Ayres et al., 2008; Daehler & Strong, 1994). The filled seeds from each quadrat were collected and placed into separate zip‐lock bags. Seeds were stored in 8 PSU seawater at 4°C in preparation for the common garden experiment (Liu et al., 2016).

**FIGURE 1:**
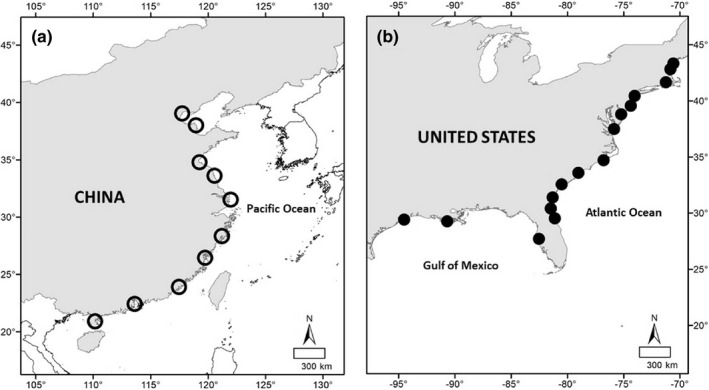
Map of *Spartina alterniflora* collection locations in the: invasive (open circles) (a), and native (closed circles) ranges (b)

### 2.2 Greenhouse common garden experiment

This study was conducted in a greenhouse common garden at Xiamen (24.62°N, 118.31°E). The mean annual temperature is 21.5℃, the sunshine duration is 1827 h/year and the relative humidity is 78%. We sampled 10 populations in China (invasive range) and 16 populations in the United States (native range). We randomly chose 10 seed families per population. We chose one seedling from each seed family (one for each of ten rectangular plastic pools: length: 1.2 m, width: 0.9 m, depth: 0.3 m), which seeds were germinated and grown in a growth chamber until seedlings were approximately 5 cm tall in March 2015, for a total of 260 plants (160 from the United States; 100 from China). One seedling was randomly assigned a position in a plastic pot (18 cm in diameter and 24 cm deep) within a block of ten blocks. Each pot contained a substrate of a mixture of peat 50% Jiffy's peat soil and 50% vermiculite by volume. Artificial sea water (10 PSU) that had been amended with fertilizer (C:N:P 15–15–15; 0.5 g per pot) to ~2 cm above the soil level in the pots was used to water the plants. The fully flooded soil in the pots could minimize variation in salinity caused by evaporation, and mimic the soil composition or the tidal regime experienced by plants in nature. Water in the pools was completely replaced once a month and salinity was checked every other day and freshwater was added as needed to maintain salinity as in Liu, Chen, et al. (2020) and Liu, Zhang, et al. (2020). From May to the end of the growing season in October 2015, we recorded the date on which the first *S*. *alterniflora* shoot flowered in each pot. In October 2015, all aboveground and belowground biomass was then harvested and oven‐dried at 60°C for 72 h and subsequently weighted. The belowground samples of each pot were gently washed over a 2‐mm mesh sieve to remove the soil substrate.

### 2.3 Statistical analyses

We used two‐sample *t*‐tests to test for differences in FFD, AGB, BGB, and BGB:AGB between the invasive and native ranges in the common garden. Data were log(x)‐transformed or square‐root(x)‐transformed or arcsin(x)‐transformed when necessary and used Shapiro–Wilk's *test* and Levene's *test* to test the normality of errors and homogeneity of variance. We used linear regression to analyze the relationships between plant traits (FFD, AGB, BGB, and BGB:AGB) and latitude of origin in the common garden. To confirm the differences in latitudinal clines between ranges, we used general linear models, with range and latitude as main factors, to determine the main and interacting effects on FFD, AGB, BGB, and BGB:AGB ratio. To confirm the effect of flowering time on biomass allocation, we used general linear models, with range and FFD as main factors, to determine the main and interacting effects on AGB, BGB, and BGB:AGB ratio. We performed all analyses using R statistical software (R Development Core Team, R version 3.6.2, 2019).

## 3 RESULTS

### 3.1 Flowering and biomass variation between ranges

**FIGURE 2:**
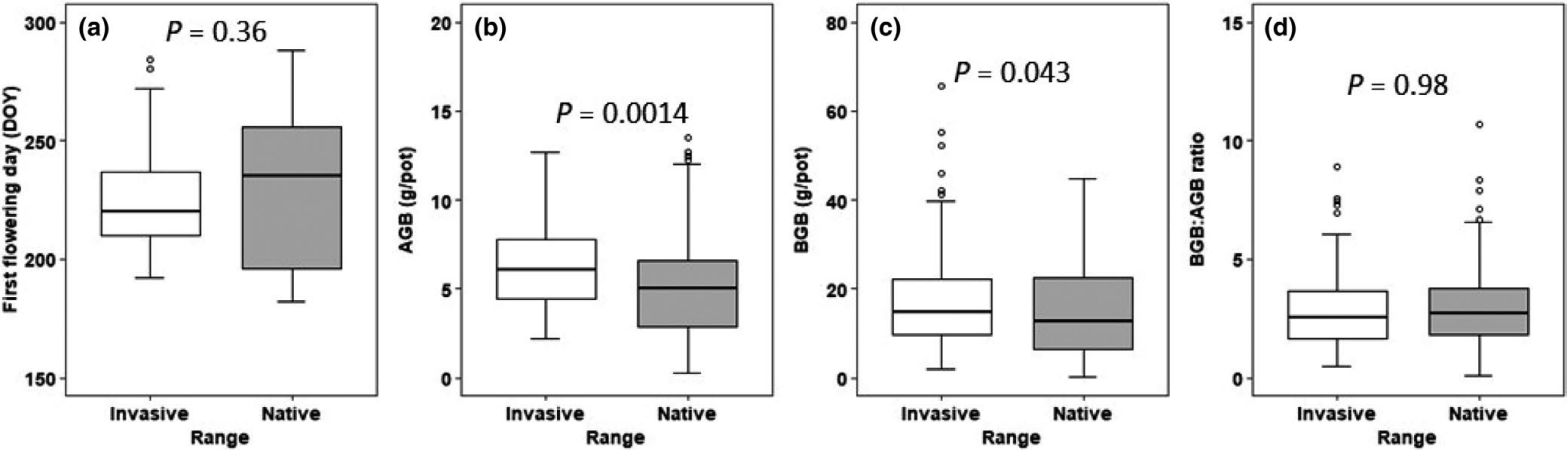
First flowering day (a), aboveground biomass (b), belowground biomass (c). and belowground biomass:aboveground biomass ratio (d) of *Spartina alterniflora* populations from the invasive (China) and native (United States) ranges in the greenhouse common garden. DOY, day of year. *p* value indicates significance of *t*‐tests between the invasive and native range

AGB and BGB of the invasive plants were on average significantly higher (~20%, ~19%) than those of the native populations (Figure 2b,c; Table 1b,c). However, there were no differences in the FFD and BGB:AGB ratio between ranges (Figure 2a,d; Table 1a,d).

**TABLE 1:** General linear models, with range and latitude as main factors, was used to determine the main and interacting effects on FFD, AGB, BGB, and BGB:AGB ratio

Traits	Factor effects	*df*	*F*	*p*
(a) FFD	Range	1	2.55	.11
	Latitude	1	133.66	**<.0001**
	Range * Latitude	1	58.64	**<.0001**
	Residuals	171		
(b) AGB	Range	1	12.02	.**0006**
	Latitude	1	23.41	**<.0001**
	Range * Latitude	1	75.67	**<.0001**
	Residuals	246		
(c) BGB	Range	1	4.59	.**03**
	Latitude	1	0.51	.47
	Range * Latitude	1	12.85	.**0004**
	Residuals	245		
(d) BGB:AGB ratio	Range	1	0.01	.96
	Latitude	1	9.57	.**002**
	Latitude*Range	1	5.52	.**02**
	Residuals	245		

Note

Entries in bold indicate statistically significantly results (*p* < .05).

Abbreviations: AGB, aboveground biomass; BGB, belowground biomass; FFD, the first flowering day.

### 3.2 Flowering and biomass variation across latitudes

**FIGURE 3:**
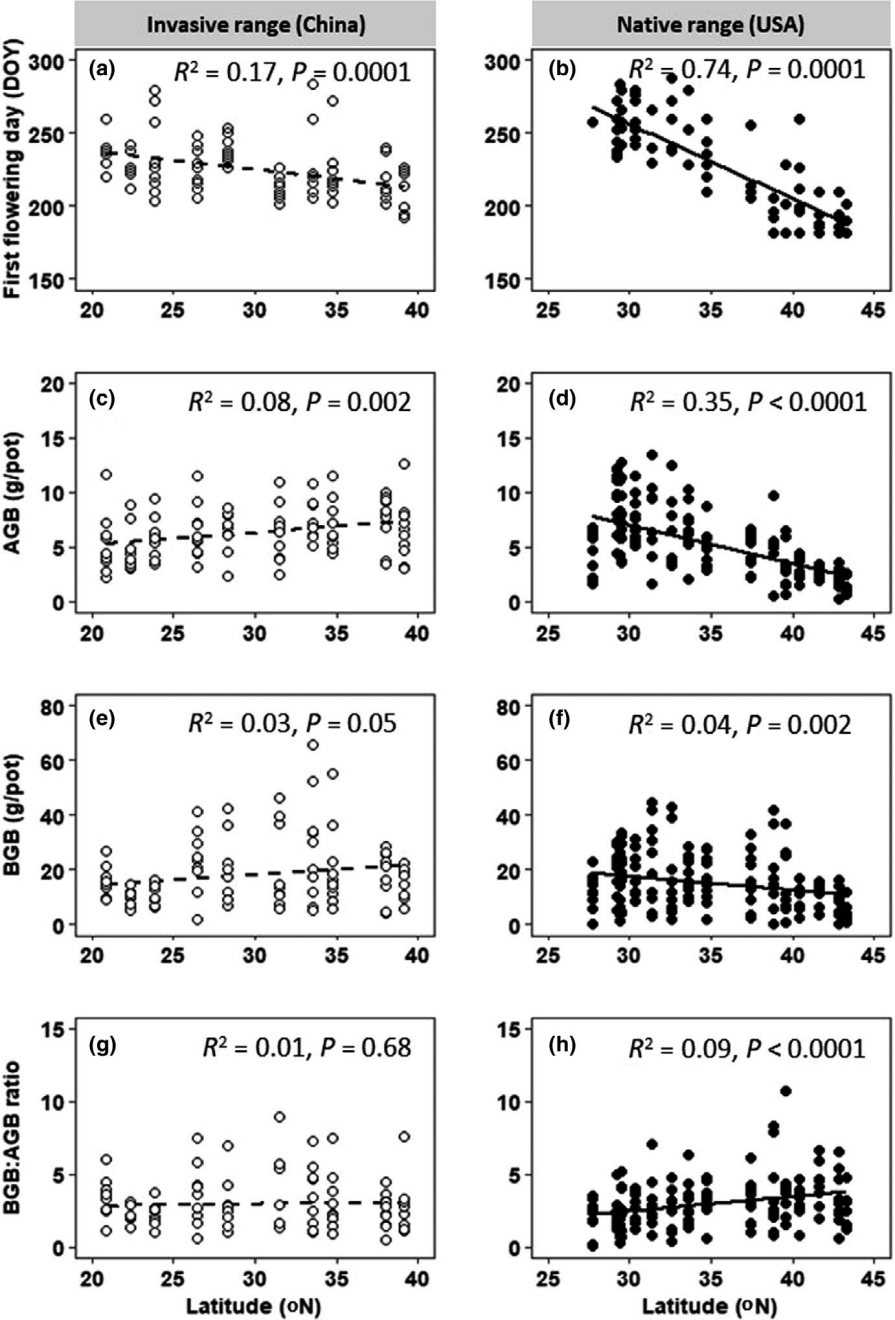
Relationships between first flowering day (a, b), aboveground biomass (c, d), belowground biomass (e, f) and belowground biomass:aboveground biomass ratio (g, h), and latitude of origin in the greenhouse common garden. DOY, day of year

In both ranges, the FFD decreased significantly with latitude, with a stronger cline in the native range (Figure 3a,b). AGB and BGB from the invasive range showed weakly positive relationships with latitude, but there was no significant relationship between BGB:AGB ratio and latitude of origin (Figure 3c,e,g). However, AGB and BGB of *S*. *alterniflora* populations from the native range significantly decreased with latitude of origin, although BGB:AGB ratio increased with latitude of origin (Figure 3d,f,h). The general linear model analyses revealed significant latitude‐by‐range interaction effects on FFD, AGB, BGB, and BGB:AGB (Table 1a–d).

### 3.3 Effects of flowering time on biomass allocation

**FIGURE 4:**
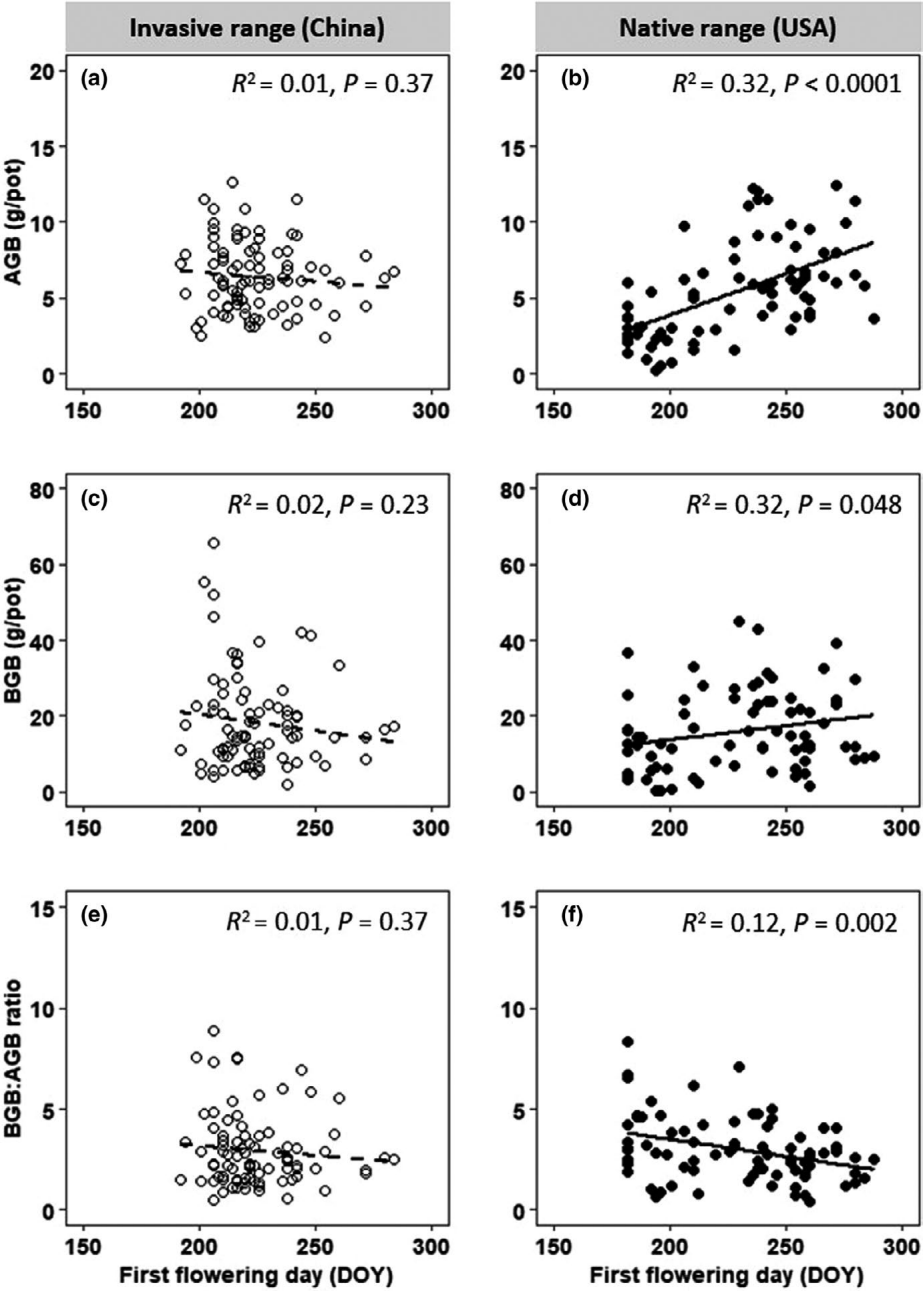
Relationships between aboveground biomass (a, b), belowground biomass (c, d) and belowground biomass:aboveground biomass ratio (e, f), and first flowering day in the greenhouse common garden. DOY, day of year

In the invasive range, AGB and BGB weakly decreased with FFD (Figure 4a,c). In contrast, in the native range, AGB and BGB strongly increased with FFD (Figure 4b,d). In both ranges, BGB:AGB ratio decreased with FFD (Figure 4e,f). FFD has a significant effect on AGB and BGB:AGB ratio (Table 2a,c), and there were significant interactions between FFD and range effect on AGB and BGB (Table 2a,b).

**TABLE 2:** General linear models, with range and FFD as main factors, was used to determine the main and interacting effects on AGB, BGB, and BGB:AGB ratio.

Traits	Factor effects	*df*	*F*	*P*
(a) AGB	Range	1	5.77	.**02**
	FFD	1	25.41	**<.0001**
	Range * FFD	1	16.75	**<.0001**
	Residuals	171		
(b) BGB	Range	1	1.78	.18
	FFD	1	0.75	.39
	Range * FFD	1	4.16	.**04**
	Residuals	170		
(c) BGB:AGB ratio	Range	1	0.07	.79
	FFD	1	8.69	.**004**
	Range * FFD	1	0.44	.51
	Residuals	170		

Note

Entries in bold indicate statistically significantly results (*p* < .05).

Abbreviations: AGB, aboveground biomass; BGB, belowground biomass; FFD, the first flowering day.

## 4 DISCUSSION

Salt marshes play an important role in carbon sequestration globally and provide numerous ecosystem services (Barbier et al., 2011). Many of these services stem from the productivity of salt marsh systems (Kirwan & Murray, 2007). Our data suggest that changes in FFD and biomass allocation with latitude are key processes in productivity of salt marshes. In comparing growth patterns and life‐history traits of *S*. *alterniflora* plants grown in a common garden, we found that invasive populations produced more AGB and BGB biomass than native populations, supporting the EICA hypothesis, in which invasive plants escape from their native enemies and can, therefore, divert resources from defense to growth, improving their competitive ability (Blossey & Nötzold, 1995; Bossdorf et al., 2005; Maron et al., 2004). However, we did not find any significant differences in FFD and BGB:AGB ratio between ranges, indicating that climatic conditions in the native range of species selected for a flowering and allocation pattern are maintained after being introduced to the invasive range (Crosby et al., 2015; Godoy et al., 2009; Shang et al., 2015). And the environmental conditions introduced in China along latitudes are similar to the native range where *S*. *alterniflora* evolved (Kirwan et al., 2009; Liu, Chen, et al., 2020; Liu, Zhang, et al., 2020). Moreover, our results demonstrated that parallel flowering time clines across latitudes resulted in contrasting AGB and BGB clines between introduced and native populations, indicating invasive populations may have broken free of the genetic constraints predicted by life‐history trade‐offs (Colautti & Barrett, 2010). We also found flowering time negatively correlated (*p* = .002) with BGB:AGB ratio in the native range, indicating earlier flowering time would shift biomass allocation from above‐ to belowground (Crosby et al., 2015; Woods et al., 2012). *S*. *alterniflora* can evolve parallel or different latitudinal clines rapidly in invasive and native ranges, with implications for further the range expansion across latitude (Colautti & Barrett, 2010).

Plants often perform better in their invasive ranges compared to in their native ranges, showing invasive phenotypes such as larger size, and higher biomass (Bossdorf et al., 2005; Colautti et al., 2009; Hierro et al., 2005; Hodgins et al., 2018; van Kleunen et al., 2010, 2018; Leger & Rice, 2003) and earlier flowering at times depending on the local environment in the introduced range (McGoey et al., 2020). Our results showed that the AGB and BGB were higher in the invasive plants than in the native plants in the greenhouse common garden, consistent with previous studies on *S*. *alterniflora* (Liu, Chen, et al., 2020; Liu, Zhang, et al., 2020; Qing et al., 2011; Shang et al., 2015). *S*. *alterniflora* suffers higher herbivory pressure in its native than in its invasive range (Gratton & Denno, 2005; Holdredge et al., 2009; Li et al., 2009; Silliman et al., 2005). So, the invasive plants could be re‐directing resources from herbivore defense to growth. Intraspecific hybrid vigor is thought to have played a role in the spread of other invasive species (Glotzbecker et al., 2016). Genetic admixture can facilitate colonization via hybrid vigor and profoundly enhance invasion via contributing novel genetic variation to adaption (Rius & Darling, 2014). *S*. *alterniflora* in China had multiple origins from three US provenances, which were cultivated together and crossed, and the most vigorous lineages were propagated and grew bigger (Qiao et al., 2019; Qing et al., 2011; Shang et al., 2015). However, we did not find significant differences in flowering time and BGB:AGB ratio between invasive and native ranges, which indicated flowering and the biomass allocation between above‐ and belowground are the results of local climate adaptation in the native range similar to the invasive range (Chen et al., 2021; Crosby et al., 2015; Liu, Chen, et al., 2020; Liu, Zhang, et al., 2020).

Flowering time is a key life‐history trait that can have a major impact on fitness and is thus likely a target of strong selection during invasion and spread across large geographic areas (Colautti & Barrett, 2010; Weiner, 2004). We found flowering time advanced with increasing latitude in both invasive and native populations, which is consistent with invasive populations being pre‐adapted to latitudinal clines from the native range (Colautti & Barrett, 2013; Hodgins & Rieseberg, 2011; Leger & Rice, 2007; Montague et al., 2008; Samis et al., 2012; Stinchcombe et al., 2004). This cline may be caused by the more compressed growing season with increasing latitude, leading to earlier flowering times at high latitudes compared to lower ones (Novy et al., 2013; Pau et al., 2011). Because all the *S*. *alterniflora* plants in China had a common origin from seeds of genetic admixtures among three native provenance four decades ago (Qiao et al., 2019), this indicates rapid and perhaps ongoing selection for earlier flowering times at sites with a shorter growing season, as occurs in the native range (Crosby et al., 2015; Seneca, 1974; Somers & Grant, 1981).

Previous work has found that aboveground biomass showed a hump‐shaped relationship with latitude in the field in the invasive range, and that these patterns were largely due to phenotypic plasticity (Liu, Chen, et al., 2020). However, this study did not quantify belowground biomass and biomass allocation, and so could not establish whether it follows the same latitudinal patterns as aboveground biomass. In the greenhouse common garden, AGB and BGB decreased with latitude in the native range, but weakly increased with latitude in the invasive range, and it has been proved that patterns disappeared in the second year (Liu, Chen, et al., 2020). This suggests that the AGB and BGB clines across latitudes are driven by a genetic basis in the native range, but mainly by phenotypic plasticity in the invasive ranges (Liu, Chen, et al., 2020). In North America, latitudinal patterns in *S*. *alterniflora* traits often have a strong genetic component (Seliskar et al., 2002; Travis & Grace, 2010), supporting the genetic differentiation of different traits (Blum et al., 2007; Qiao et al., 2019; Strong & Ayres, 2013). However, while the BGB:AGB ratio increased with latitude in the native range, this cline disappeared in the invasive range, indicating that invasive populations have freed them from the trade‐off between these two competing modes as seen in the native range (Crosby et al., 2015). Otherwise, with populations at higher latitudes experiencing less severe competition in the invasive range because of largely vacant niches with fewer conspecifics in mudflat habitats, weak selective pressure for competitive ability (vegetative growth) permitted plants to invest less energy in aboveground biomass and more energy in belowground biomass (Bertness & Hacker, 1994; Bhattarai et al., 2017; Schemske et al., 2009).

Shifting in biomass allocation from above‐ to belowground was related to flowering phenology (Crosby et al., 2015). We found that first flowering day positively correlated with AGB and BGB in the native range, where northern populations flower earlier at a smaller size compared with southern populations, consistent with an adaptive response to latitudinal changes in growing‐season length and life‐history trade‐offs (Chen et al., 2021; Colautti & Barrett, 2010). In contrast to native range, the first flowering time negatively correlated with AGB and BGB in the invasive range, indicating that natural selection will favor genotypes with both earlier flowering time and large size because of higher fitness, in conflict with the life‐history paradigm relating flowering time and plant size (Colautti & Barrett, 2010). Moreover, we found that flowering time positively correlated with BGB:AGB ratio in both native and invasive range, indicating that flowering phenology would be related to the biomass allocation of plants (Crosby et al., 2015; Weiner, 2004). Our results are consistent with previous findings that higher root‐to‐shoot ratios were genetically correlated with early phenology (Woods et al., 2012). Therefore, belowground biomass accumulation will be affected by the start, end, and length of the growing season (Crosby et al., 2015; Liu et al., 2022). Future predictions of salt marsh growth and accretion should thus consider not only the impact of the length of the growing season but also the plant's life cycle (Weiner, 2004). In general, our results suggest that flowering time is the primary driver of the aboveground versus belowground biomass allocation pattern for plants (Cheng et al., 2015). One caveat of this study is that all plants were grown from seed and, therefore, subject to maternal effects. However, we think it unlikely that maternal effects could explain the phenotypic patterns presented here because maternal effects are more pervasive in early life‐history stages (Liu, Chen, et al., 2020; Liu, Zhang, et al., 2020; Montague et al., 2008; Rossiter, 1998), while here we focused on traits at the end of the annual life cycle.

This study has shown that *S*. *alterniflora* would produce more AGB and BGB in the invasive range than in the native range, which is consistent with EICA hypothesis. This study also has identified parallel flowering time clines in the invasive and native ranges, but contrasting AGB and BGB clines. One of the more significant findings to emerge from this study is that invasive populations have broken free of the genetic constrains relating earlier flowering with smaller size. Larger size at earlier flowering time will contribute to the enhancement and evolution in competitive ability, and further result in higher reproductive output and fitness, which will facilitate the evolutionary response of invasive species to local environment and further the range expansion (Colautti & Barrett, 2010). Given that, we recover negative relationships between flowering time and AGB and BGB in the invasive range. Moreover, earlier flowering would shift aboveground biomass to belowground biomass. These findings have significant implications for our understanding of how flowering phenology affects biomass allocation across latitudes. When we model the productivity of salt marshes, we should, therefore, consider the flowering phenology as an important cue in the future. Finally, we propose that rapid evolution, biomass reallocation across latitudes and phenotypic plasticity of this invasive species contribute to successful invasion of plants.

## CONFLICT OF INTEREST

The authors declare that there is no conflict of interest regarding the publication of this article.

## AUTHOR CONTRIBUTIONS

**Wenwen Liu:** Funding acquisition (equal); Investigation (equal); Writing – original draft (lead); Writing – review & editing (equal). **Xincong Chen:** Investigation (equal); Writing – original draft (equal); Writing – review & editing (equal). **Jiayu Wang:** Investigation (supporting); Software (equal); Writing – original draft (supporting); Writing – review & editing (supporting). **Yihui Zhang:** Funding acquisition (lead); Investigation (equal); Writing – original draft (equal); Writing – review & editing (equal).

## Data Availability

The salt marsh grass *Spartina alterniflora* flowering and biomass data have been deposited in Dryad and are available at https://doi.org/10.5061/dryad.2rbnzs7q7.

## References

[ece38681-bib-0001] Alexander, J. M. , van Kleunen, M. , Ghezzi, R. , & Edwards, P. J. (2012). Different genetic clines in response to temperature across the native and introduced ranges of a global plant invader. Journal of Ecology, 100(3), 771–781. 10.1111/j.1365-2745.2011.01951.x

[ece38681-bib-0002] An, S. , Gu, B. , Zhou, C. , Wang, Z. , Deng, Z. F. , Zhi, Y. , Li, H. , Chen, L. , Yu, D. , & Liu, Y. (2007). *Spartina* invasion in China: Implications for invasive species management and future research. Weed Research, 47(3), 183–191. 10.1111/j.1365-3180.2007.00559.x

[ece38681-bib-0003] Ayres, D. R. , Zaremba, K. , Sloop, C. M. , & Strong, D. R. (2008). Sexual reproduction of cordgrass hybrids (*Spartina foliosa* x *alterniflora*) invading tidal marshes in San Francisco Bay. Diversity and Distributions, 14(2), 187–195. 10.1111/j.1472-4642.2007.00414.x

[ece38681-bib-0004] Barbier, E. B. , Hacker, S. D. , Kennedy, C. , Koch, E. W. , Stier, A. C. , & Silliman, B. R. (2011). The value of estuarine and coastal ecosystem services. Ecological Monographs, 81(2), 169–193. 10.1890/10-1510.1

[ece38681-bib-0005] Barrett, S. C. H. , Colautti, R. I. , & Eckert, C. G. (2008). Plant reproductive systems and evolution during biological invasion. Molecular Ecology, 17(1), 373–383. 10.1111/j.1365-294X.2007.03503.x 17868309

[ece38681-bib-0006] Beckmann, M. , Erfmeier, A. , & Bruelheide, H. (2009). A comparison of native and invasive populations of three clonal plant species in Germany and New Zealand. Journal of Biogeography, 36(5), 865–878. 10.1111/j.1365-2699.2008.02048.x

[ece38681-bib-0007] Bertness, M. , & Hacker, S. (1994). Physical stress and positive associations among marsh plants. American Naturalist, 144(3), 363–372. 10.1086/285681

[ece38681-bib-0008] Bhattarai, G. P. , Meyerson, L. A. , Anderson, J. , Cummings, D. , Allen, W. J. , & Cronin, J. T. (2017). Biogeography of a plant invasion: Genetic variation and plasticity in latitudinal clines for traits related to herbivory. Ecological Monographs, 87(1), 57–75. 10.1002/ecm.1233

[ece38681-bib-0009] Blossey, B. , & Nötzold, R. (1995). Evolution of increased competitive ability in invasive nonindigenous plants: A Hypothesis. Journal of Ecology, 83(5), 887–889. 10.2307/2261425

[ece38681-bib-0010] Blum, M. J. , Bando, K. J. , Katz, M. , & Strong, D. R. (2007). Geographic structure, genetic diversity and source tracking of *Spartina alterniflora* . Journal of Biogeography, 34(12), 2055–2069. 10.1111/j.1365-2699.2007.01764.x

[ece38681-bib-0011] Bossdorf, O. , Auge, H. , Lafuma, L. , Rogers, W. E. , Siemann, E. , & Prati, D. (2005). Phenotypic and genetic differentiation between native and introduced plant populations. Oecologia, 144(1), 1–11. 10.1007/s00442-005-0070-z 15891837

[ece38681-bib-0012] Brown, J. S. , & Eckert, C. G. (2005). Evolutionary increase in sexual and clonal reproductive capacity during biological invasion in an aquatic plant *Butomus umbellatus* (Butomaceae). American Journal of Botany, 92(3), 495–502. 10.3732/ajb.92.3.495 21652427

[ece38681-bib-0013] Castillo, J. M. , Gallego‐Tévar, B. , Figueroa, E. , Grewell, B. J. , Vallet, D. , Rousseau, H. , Keller, J. , Lima, O. , Dréano, S. , Salmon, A. , & Aïnouche, M. (2018). Low genetic diversity contrasts with high phenotypic variability in heptaploid *Spartina densiflora* populations invading the Pacific coast of North America. Ecology and Evolution, 8(10), 4992–5007. 10.1002/ece3.4063 29876076 PMC5980529

[ece38681-bib-0014] Castillo, J. M. , Grewell, B. J. , Pickart, A. , Bortolus, A. , Peña, C. , Figueroa, E. , & Sytsma, M. (2014). Phenotypic plasticity of invasive *Spartina densiflora* (Poaceae) along a broad latitudinal gradient on the Pacific Coast of North America. American Journal of Botany, 101(3), 448–458. 10.3732/ajb.1400014 24607513

[ece38681-bib-0015] Castillo, J. M. , Leira‐Doce, P. , Rubio‐Casal, A. E. , & Figueroa, E. (2008). Spatial and temporal variations in aboveground and belowground biomass of *Spartina maritima* (small cordgrass) in created and natural marshes. Estuarine, Coastal and Shelf Science, 78(4), 819–826. 10.1016/j.ecss.2008.02.021

[ece38681-bib-0016] Chen, X. , Liu, W. , Pennings, S. C. , & Zhang, Y. (2021). Plasticity and selection drive hump‐shaped latitudinal patterns of flowering phenology in an invasive intertidal plant. Ecology, 102(5), e03311. 10.1002/ecy.3311 33586146

[ece38681-bib-0017] Cheng, D. , Zhong, Q. , Niklas, K. J. , Ma, Y. , Yang, Y. , & Zhang, J. (2015). Isometric scaling of above‐ and below‐ground biomass at the individual and community levels in the understorey of a sub‐tropical forest. Annals of Botany, 115(2), 303–313. 10.1093/aob/mcu238 25564468 PMC4466339

[ece38681-bib-0018] Colautti, R. I. , & Barrett, S. C. H. (2010). Natural selection and genetic constraints on flowering phenology in an invasive plant. International Journal of Plant Sciences, 171(9), 960–971. 10.1086/656444

[ece38681-bib-0019] Colautti, R. I. , & Barrett, S. C. H. (2013). Rapid adaptation to climate facilitates range expansion of an invasive plant. Science, 342(6156), 364–366. 10.1126/science.1242121 24136968

[ece38681-bib-0020] Colautti, R. I. , Maron, J. L. , & Barrett, S. C. H. (2009). Common garden comparisons of native and introduced plant populations: Latitudinal clines can obscure evolutionary inferences. Evolutionary Applications, 2(2), 187–199. 10.1111/j.1752-4571.2008.00053.x 25567860 PMC3352372

[ece38681-bib-0021] Crosby, S. C. , Angermeyer, A. , Adler, J. M. , Bertness, M. D. , Deegan, L. A. , Sibinga, N. , & Leslie, H. M. (2017). *Spartina alterniflora* biomass allocation and temperature: Implications for salt marsh persistence with sea‐level rise. Estuaries and Coasts, 40(1), 213–223. 10.1007/s12237-016-0142-9

[ece38681-bib-0022] Crosby, S. C. , Ivens‐Duran, M. , Bertness, M. D. , Davey, E. , Deegan, L. A. , & Leslie, H. M. (2015). Flowering and biomass allocation in U.S. Atlantic coast *Spartina alterniflora* . American Journal of Botany, 102(5), 669–676. 10.3732/ajb.1400534 26022481

[ece38681-bib-0023] Daehler, C. C. , & Strong, D. R. (1994). Variable reproductive output among clones of *Spartina alterniflora* (Poaceae) invading San Francisco Bay, California: The influence of herbivory, pollination, and establishment site. American Journal of Botany, 81(3), 307–313. 10.1002/j.1537-2197.1994.tb15448.x

[ece38681-bib-0024] Darby, F. A. , & Turner, R. E. (2008). Below‐ and aboveground biomass of *Spartina alterniflora*: Response to nutrient addition in a Louisiana salt marsh. Estuaries and Coasts, 31(2), 326–334. 10.1007/s12237-008-9037-8

[ece38681-bib-0025] Donohue, K. (2017). Divergence in how genetic pathways respond to environments. Trends in Plant Science, 22(10), 817–819. 10.1016/j.tplants.2017.08.008 28886912

[ece38681-bib-0026] Etterson, J. R. , Toczydlowski, R. H. , Winkler, K. J. , Kirschbaum, J. A. , & McAulay, T. S. (2016). *Solidago altissima* differs with respect to ploidy frequency and clinal variation across the prairie‐forest biome border in Minnesota. American Journal of Botany, 103(1), 22–32. 10.3732/ajb.1500146 26507110

[ece38681-bib-0027] Franklin, O. , Johansson, J. , Dewar, R. C. , Dieckmann, U. , McMurtrie, R. E. , Brännström, Å. , & Dybzinski, R. (2012). Modeling carbon allocation in trees: A search for principles. Tree Physiology, 32(6), 648–666. 10.1093/treephys/tpr138 22278378

[ece38681-bib-0028] Galloway, L. F. , & Burgess, K. S. (2012). Artificial selection on flowering time: Influence on reproductive phenology across natural light environments. Journal of Ecology, 100(4), 852–861. 10.1111/j.1365-2745.2012.01967.x

[ece38681-bib-0029] Glotzbecker, G. J. , Walters, D. M. , & Blum, M. J. (2016). Rapid movement and instability of an invasive hybrid swarm. Evolutionary Applications, 9(6), 741–755. 10.1111/eva.12371 27330551 PMC4908461

[ece38681-bib-0030] Godoy, O. , Castro‐Diez, P. , Valladares, F. , & Costa‐Tenorio, M. (2009). Different flowering phenology of alien invasive species in Spain: Evidence for the use of an empty temporal niche? Plant Biology, 11(6), 803–811. 10.1111/j.1438-8677.2008.00185.x 19796357

[ece38681-bib-0031] Gratton, C. , & Denno, R. F. (2005). Restoration of arthropod assemblages in a *Spartina* salt marsh following removal of the invasive plant *Phragmites australis* . Restoration Ecology, 13(2), 358–372. 10.1111/j.1526-100X.2005.00045.x

[ece38681-bib-0032] Griffith, T. M. , & Watson, M. A. (2005). Stress avoidance in a common annual: Reproductive timing is important for local adaptation and geographic distribution. Journal of Evolutionary Biology, 18(6), 1601–1612. 10.1111/j.1420-9101.2005.01021.x 16313471

[ece38681-bib-0033] Griffith, T. M. , & Watson, M. A. (2006). Is evolution necessary for range expansion? Manipulating reproductive timing of a weedy annual transplanted beyond its range. American Naturalist, 167(2), 153–164. 10.1086/498945 16670977

[ece38681-bib-0034] Haggerty, B. P. , & Galloway, L. F. (2011). Response of individual components of reproductive phenology to growing season length in a monocarpic herb. Journal of Ecology, 99(1), 242–253. 10.1111/j.1365-2745.2010.01744.x

[ece38681-bib-0035] Hanley, M. E. (2012). Seedling defoliation, plant growth and flowering potential in native‐and invasive‐range *Plantago lanceolata* populations. Weed Research, 52(3), 252–259. 10.1111/j.1365-3180.2012.00910.x

[ece38681-bib-0036] Helliwell, E. E. , Faber‐Hammond, J. , Lopez, Z. C. , Garoutte, A. , von Wettberg, E. , Friesen, M. L. , & Porter, S. S. (2018). Rapid establishment of a flowering cline in *Medicago polymorpha* after invasion of North America. Molecular Ecology, 27(23), 4758–4774. 10.1111/mec.14898 30325569

[ece38681-bib-0037] Helsen, K. , Acharya, K. P. , Graae, B. J. , De Kort, H. , Brunet, J. , Chabrerie, O. , Cousins, A. O. , De Frenne, P. D. , Hermy, M. , Verheyen, K. , & Pélabon, C. (2020). Earlier onset of flowering and increased reproductive allocation of an annual invasive plant in the north of its novel range. Annals of Botany, 126(6), 1005–1016. 10.1093/aob/mcaa110 32582950 PMC7596373

[ece38681-bib-0038] Hierro, J. L. , Maron, J. L. , & Callaway, R. M. (2005). A biogeographical approach to plant invasions: The importance of studying exotics in their introduced and native range. Journal of Ecology, 93(1), 5–15. 10.1111/j.0022-0477.2004.00953.x

[ece38681-bib-0039] Hodgins, K. A. , Bock, D. G. , & Rieseberg, L. H. (2018). Trait evolution in invasive species. In: Annual plant reviews online (pp. 459–496). American Cancer Society. 10.1002/9781119312994.apr0643

[ece38681-bib-0040] Hodgins, K. A. , & Rieseberg, L. (2011). Genetic differentiation in life‐history traits of introduced and native common ragweed (*Ambrosia artemisiifolia*) populations. Journal of Evolutionary Biology, 24(12), 2731–2749. 10.1111/j.1420-9101.2011.02404.x 22023052

[ece38681-bib-0041] Holdredge, C. , Bertness, M. D. , & Altieri, A. H. (2009). Role of crab herbivory in die‐off of New England salt marshes. Conservation Biology, 23(3), 672–679. 10.1111/j.1523-1739.2008.01137.x 19183205

[ece38681-bib-0042] Kirwan, M. L. , Guntenspergen, G. R. , & Morris, J. T. (2009). Latitudinal trends in *Spartina alterniflora* productivity and the response of coastal marshes to global change. Global Change Biology, 15(8), 1982–1989. 10.1111/j.1365-2486.2008.01834.x

[ece38681-bib-0043] Kirwan, M. L. , & Megonigal, J. P. (2013). Tidal wetland stability in the face of human impacts and sea‐level rise. Nature, 504(7478), 53–60. 10.1038/nature12856 24305148

[ece38681-bib-0044] Kirwan, M. L. , & Mudd, S. M. (2012). Response of salt‐marsh carbon accumulation to climate change. Nature, 489(7417), 550–553. 10.1038/nature11440 23018965

[ece38681-bib-0045] Kirwan, M. L. , & Murray, A. B. (2007). A coupled geomorphic and ecological model of tidal marsh evolution. Proceedings of the National Academy of Sciences of the United States of America, 104(15), 6118–6122. 10.1073/pnas.0700958104 17389384 PMC1851060

[ece38681-bib-0046] Kollmann, J. , & Banuelos, M. J. (2004). Latitudinal trends in growth and phenology of the invasive alien plant *Impatiens glandulifera* (Balsaminaceae). Diversity and Distributions, 10(5–6), 377–385. 10.1111/j.1366-9516.2004.00126.x

[ece38681-bib-0047] Leger, E. A. , & Rice, K. J. (2003). Invasive California poppies (*Eschscholzia californica* Cham.) grow larger than native individuals under reduced competition. Ecology Letters, 6(3), 257–264. 10.1046/j.1461-0248.2003.00423.x

[ece38681-bib-0048] Leger, E. A. , & Rice, K. J. (2007). Assessing the speed and predictability of local adaptation in invasive California poppies (*Eschscholzia californica*). Journal of Evolutionary Biology, 20(3), 1090–1103. 10.1111/j.1420-9101.2006.01292.x 17465919

[ece38681-bib-0049] Li, B. , Liao, C. , Zhang, X. , Chen, H. , Wang, Q. , Chen, Z. , Gan, X. , Wu, J. , Zhao, B. , Ma, Z. , Cheng, X. , Jiang, L. , & Chen, J. (2009). *Spartina alterniflora* invasions in the Yangtze River estuary, China: An overview of current status and ecosystem effects. Ecological Engineering, 35(4), 511–520. 10.1016/j.ecoleng.2008.05.013

[ece38681-bib-0050] Li, X. , She, D. , Zhang, D. , & Liao, W. (2015). Life history trait differentiation and local adaptation in invasive populations of *Ambrosia artemisiifolia* in China. Oecologia, 177, 669–677. 10.1007/s00442-014-3127-z 25362583

[ece38681-bib-0051] Liu, H. , Wang, H. , Li, N. , Shao, J. , Zhou, X. , van Groenigen, K. J. , & Thakur, M. P. (2022). Phenological mismatches between above‐and belowground plant responses to climate warming. Nature Climate Change, 12, 97–102. 10.1038/s41558-021-01244-x

[ece38681-bib-0052] Liu, W. , Chen, X. , Strong, D. R. , Pennings, S. C. , Kirwan, M. L. , Chen, X. , & Zhang, Y. (2020). Climate and geographic adaptation drive latitudinal clines in biomass of a widespread saltmarsh plant in its native and introduced ranges. Limnology and Oceanography, 65(6), 1399–1409. 10.1002/lno.11395

[ece38681-bib-0053] Liu, W. , Maung‐Douglass, K. , Strong, D. R. , Pennings, S. C. , & Zhang, Y. (2016). Geographical variation in vegetative growth and sexual reproduction of the invasive *Spartina alterniflora* in China. Journal of Ecology, 104(1), 173–181. 10.1111/1365-2745.12487

[ece38681-bib-0054] Liu, W. , & Pennings, S. C. (2019). Self‐thinning and size‐dependent flowering of the grass *Spartina alterniflora* across space and time. Functional Ecology, 33(10), 1830–1841. 10.1111/1365-2435.13384

[ece38681-bib-0055] Liu, W. , Strong, D. R. , Pennings, S. C. , & Zhang, Y. (2017). Provenance‐by‐environment interaction of reproductive traits in the invasion of *Spartina alterniflora* in China. Ecology, 98(6), 1591–1599. 10.1002/ecy.1815 28316076

[ece38681-bib-0056] Liu, W. , Zhang, Y. , Chen, X. , Maung‐Douglass, K. , Strong, D. R. , & Pennings, S. C. (2020). Contrasting plant adaptation strategies to latitude in the native and invasive range of *Spartina alterniflora* . New Phytologist, 226(2), 623–634. 10.1111/nph.16371 31834631

[ece38681-bib-0057] Ma, H. , Mo, L. , Crowther, T. W. , Maynard, D. S. , van den Hoogen, J. , Stocker, B. D. , Terrer, C. , & Zohner, C. M. (2021). The global distribution and environmental drivers of aboveground versus belowground plant biomass. Nature Ecology & Evolution, 5(8), 1110–1122. 10.1038/s41559-021-01485-1 34168336

[ece38681-bib-0058] Maron, J. L. , Vila, M. , Bommarco, R. , Elmendorf, S. , & Beardsley, P. (2004). Rapid evolution of an invasive plant. Ecological Monographs, 74(2), 261–280. 10.1890/03-4027

[ece38681-bib-0059] McGoey, B. V. , Hodgins, K. A. , & Stinchcombe, J. R. (2020). Parallel flowering time clines in native and introduced ragweed populations are likely due to adaptation. Ecology and Evolution, 10(11), 4595–4608. 10.1002/ece3.6163 32551046 PMC7297792

[ece38681-bib-0060] Mcleod, E. , Chmura, G. L. , Bouillon, S. , Salm, R. , Bjork, M. , Duarte, C. M. , Lovelock, C. E. , Schlesinger, W. H. , & Silliman, B. R. (2011). A blueprint for blue carbon: Toward an improved understanding of the role of vegetated coastal habitats in sequestering CO_2_ . Frontiers in Ecology and the Environment, 9(10), 552–560. 10.1890/110004

[ece38681-bib-0061] Meyer, G. A. , & Hull‐Sanders, H. M. (2008). Altered patterns of growth, physiology and reproduction in invasive genotypes of *Solidago gigantea* (Asteraceae). Biological Invasions, 10(3), 303–317. 10.1007/s10530-007-9131-z

[ece38681-bib-0062] Montague, J. L. , Barrett, S. C. H. , & Eckert, C. G. (2008). Re‐establishment of clinal variation in flowering time among introduced populations of purple loosestrife (*Lythrum salicaria*, Lythraceae). Journal of Evolutionary Biology, 21(1), 234–245. 10.1111/j.1420-9101.2007.01456.x 18028354

[ece38681-bib-0063] Novy, A. , Flory, S. L. , & Hartman, J. M. (2013). Evidence for rapid evolution of phenology in an invasive grass. Journal of Evolutionary Biology, 26(2), 443–450. 10.1111/jeb.12047 23194053

[ece38681-bib-0064] Pau, S. , Wolkovich, E. M. , Cook, B. I. , Davies, T. J. , Kraft, N. J. B. , Bolmgren, K. , Betancourt, J. L. , & Cleland, E. E. (2011). Predicting phenology by integrating ecology, evolution and climate science. Global Change Biology, 17(12), 3633–3643. 10.1111/j.1365-2486.2011.02515.x

[ece38681-bib-0065] Qiao, H. , Liu, W. , Zhang, Y. , Zhang, Y.‐Y. , & Li, Q. Q. (2019). Genetic admixture accelerates invasion via provisioning rapid adaptive evolution. Molecular Ecology, 28(17), 4012–4027. 10.1111/mec.15192 31339595

[ece38681-bib-0066] Qing, H. , Yao, Y. , Xiao, Y. , Hu, F. , Sun, Y. , Zhou, C. , & An, S. (2011). Invasive and native tall forms of *Spartina alterniflora* respond differently to nitrogen availability. Acta Oecologica‐International Journal of Ecology, 37(1), 23–30. 10.1016/j.actao.2010.11.002

[ece38681-bib-0067] Ridley, C. E. , & Ellstrand, N. C. (2010). Rapid evolution of morphology and adaptive life history in the invasive California wild radish (*Raphanus sativus*) and the implications for management. Evolutionary Applications, 3(1), 64–76. 10.1111/j.1752-4571.2009.00099.x 25567904 PMC3352453

[ece38681-bib-0068] Rius, M. , & Darling, J. A. (2014). How important is intraspecific genetic admixture to the success of colonising populations? Trends in Ecology & Evolution, 29(4), 233–242. 10.1016/j.tree.2014.02.003 24636862

[ece38681-bib-0069] Rossiter, M. (1998). Assessment of genetic variation in the presence of maternal or paternal effects in herbivorous insects. In S. Mopper , & S. Y. Strauss (Eds.), In Genetic structure and local adaptation in natural insect populations: Effects of ecology, life history, and behavior (pp. 113–138). Springer. 10.1007/978-1-4757-0902-5_6

[ece38681-bib-0070] Samis, K. E. , Murren, C. J. , Bossdorf, O. , Donohue, K. , Fenster, C. B. , Malmberg, R. L. , Purugganan, M. D. , & Stinchcombe, J. R. (2012). Longitudinal trends in climate drive flowering time clines in North American *Arabidopsis thaliana* . Ecology and Evolution, 2(6), 1162–1180. 10.1002/ece3.262 22833792 PMC3402192

[ece38681-bib-0071] Santamaria, L. , Figuerola, J. , Pilon, J. J. , Mjelde, M. , Green, A. J. , De Boer, T. , King, R. A. , & Gornall, R. J. (2003). Plant performance across latitude: The role of plasticity and local adaptation in an aquatic plant. Ecology, 84(9), 2454–2461. 10.1890/02-0431

[ece38681-bib-0072] Schemske, D. W. , Mittelbach, G. G. , Cornell, H. V. , Sobel, J. M. , & Roy, K. (2009). Is there a latitudinal gradient in the importance of biotic interactions? Annual Review of Ecology Evolution and Systematics, 40, 245–269. 10.1146/annurev.ecolsys.39.110707.173430

[ece38681-bib-0073] Seliskar, D. M. , Gallagher, J. L. , Burdick, D. M. , & Mutz, L. A. (2002). The regulation of ecosystem functions by ecotypic variation in the dominant plant: A *Spartina alterniflora* salt‐marsh case study. Journal of Ecology, 90(1), 1–11. 10.1046/j.0022-0477.2001.00632.x

[ece38681-bib-0074] Seneca, E. D. (1974). Germination and seedling response of Atlantic and Gulf coasts populations of *Spartina alterniflora* . American Journal of Botany, 61(9), 947–956. 10.1002/j.1537-2197.1974.tb14034.x

[ece38681-bib-0075] Shang, L. , Qiu, S. , Huang, J. , & Li, B. (2015). Invasion of *Spartina alterniflora* in China is greatly facilitated by increased growth and clonality: A comparative study of native and introduced populations. Biological Invasions, 17(5), 1327–1339. 10.1007/s10530-014-0796-9

[ece38681-bib-0076] Silliman, B. R. , van de Koppel, J. , Bertness, M. D. , Stanton, L. E. , & Mendelssohn, I. A. (2005). Drought, snails, and large‐scale die‐off of southern US salt marshes. Science, 310(5755), 1803–1806. 10.1126/science.1118229 16357258

[ece38681-bib-0077] Snedden, G. A. , Cretini, K. , & Patton, B. (2015). Inundation and salinity impacts to above‐ and belowground productivity in *Spartina patens* and *Spartina alterniflora* in the Mississippi River deltaic plain: Implications for using river diversions as restoration tools. Ecological Engineering, 81, 133–139. 10.1016/j.ecoleng.2015.04.035

[ece38681-bib-0078] Sobrinho, M. S. , Tabatinga, G. M. , Machado, I. C. , & Lopes, A. V. (2013). Reproductive phenological pattern of *Calotropis procera* (Apocynaceae), an invasive species in Brazil: Annual in native areas; continuous in invaded areas of caatinga. Acta Botanica Brasilica, 27, 456–459. 10.1590/S0102-33062013000200018

[ece38681-bib-0079] Somers, G. , & Grant, D. (1981). Influence of seed source upon phenology of flowering of *Spartina alterniflora* Loisel. And the likelihood of cross pollination. American Journal of Botany, 68(1), 6–9. 10.2307/2442985

[ece38681-bib-0080] Stearns, S. C. (1989). Trade‐offs in life‐history evolution. Functional Ecology, 3(3), 259–268. 10.2307/2389364

[ece38681-bib-0081] Stinchcombe, J. R. , Weinig, C. , Ungerer, M. , Olsen, K. M. , Mays, C. , Halldorsdottir, S. S. , Purugganan, M. D. , & Schmitt, J. (2004). A latitudinal cline in flowering time in *Arabidopsis thaliana* modulated by the flowering time gene FRIGIDA. Proceedings of the National Academy of Sciences of the United States of America, 101(13), 4712–4717. 10.1073/pnas.0306401101 15070783 PMC384812

[ece38681-bib-0082] Strong, D. R. , & Ayres, D. R. (2013). Ecological and evolutionary misadventures of *Spartina* . Annual Review of Ecology, Evolution, and Systematics, 44, 389–410. 10.1146/annurev-ecolsys-110512-135803

[ece38681-bib-0083] Sun, Y. , & Roderick, G. K. (2019). Rapid evolution of invasive traits facilitates the invasion of common ragweed, *Ambrosia artemisiifolia* . Journal of Ecology, 107(6), 2673–2687. 10.1111/1365-2745.13198

[ece38681-bib-0084] Townend, I. , Fletcher, C. , Knappen, M. , & Rossington, K. (2011). A review of salt marsh dynamics. Water and Environment Journal, 25(4), 477–488. 10.1111/j.1747-6593.2010.00243.x

[ece38681-bib-0085] Travis, S. E. , & Grace, J. B. (2010). Predicting performance for ecological restoration: A case study using *Spartina alternfliora* . Ecological Applications, 20(1), 192–204. 10.1890/08-1443.1 20349840

[ece38681-bib-0086] van Boheemen, L. A. , Atwater, D. Z. , & Hodgins, K. A. (2019). Rapid and repeated local adaptation to climate in an invasive plant. New Phytologist, 222(1), 614–627. 10.1111/nph.15564 30367474

[ece38681-bib-0087] van Kleunen, M. , Bossdorf, O. , & Dawson, W. (2018). The ecology and evolution of alien plants. Annual Review of Ecology, Evolution, and Systematics, 49(1), 25–47. 10.1146/annurev-ecolsys-110617-062654

[ece38681-bib-0088] van Kleunen, M. , Weber, E. , & Fischer, M. (2010). A meta‐analysis of trait differences between invasive and non‐invasive plant species. Ecology Letters, 13(2), 235–245. 10.1111/j.1461-0248.2009.01418.x 20002494

[ece38681-bib-0089] Weiner, J. (2004). Allocation, plasticity and allometry in plants. Perspectives in Plant Ecology Evolution and Systematics, 6(4), 207–215. 10.1078/1433-8319-00083

[ece38681-bib-0090] Woods, E. C. , Hastings, A. P. , Turley, N. E. , Heard, S. B. , & Agrawal, A. A. (2012). Adaptive geographical clines in the growth and defense of a native plant. Ecological Monographs, 82(2), 149–168. 10.1890/11-1446.1

[ece38681-bib-0091] Xu, G. , & Zhuo, R. (1985). Preliminary studies of introduced *Spartina alterniflora* Loisel. in China (I) (in Chinese with English abstract). Journal of Nanjing University, 40, 212–225. https://www.researchgate.net/publication/284873712_Preliminary_studies_of_introduced_Spartina_alterniflora_Loisel_in_China_I

[ece38681-bib-0092] Zenni, R. D. , Lamy, J. B. , Lamarque, L. J. , & Porté, A. J. (2014). Adaptive evolution and phenotypic plasticity during naturalization and spread of invasive species: implications for tree invasion biology. Biological Invasions, 16(3), 635–644. 10.1007/s10530-013-0607-8

